# Microfabricated Engineered Particle Systems for Respiratory Drug Delivery and Other Pharmaceutical Applications

**DOI:** 10.1155/2012/941243

**Published:** 2012-02-09

**Authors:** Andres Garcia, Peter Mack, Stuart Williams, Catherine Fromen, Tammy Shen, Janet Tully, Jonathan Pillai, Philip Kuehl, Mary Napier, Joseph M. DeSimone, Benjamin W. Maynor

**Affiliations:** ^1^Liquidia Technologies, Research Triangle Park, NC 27709, USA; ^2^Department of Chemical & Biomolecular Engineering, North Carolina State University, Raleigh, NC 27695, USA; ^3^Eshelman School of Pharmacy, University of North Carolina at Chapel Hill, Chapel Hill, NC 27599, USA; ^4^Department of Chemistry, University of North Carolina at Chapel Hill, Chapel Hill, NC 27599, USA; ^5^Lovelace Respiratory Research Institute, 2425 Ridgecrest Dr. SE, Albuquerque, NM 87108, USA

## Abstract

Particle Replication in Non-Wetting Templates (PRINT^®^) is a platform particle drug delivery technology that coopts the precision and nanoscale spatial resolution inherently afforded by lithographic techniques derived from the microelectronics industry to produce precisely engineered particles. We describe the utility of PRINT technology as a strategy for formulation and delivery of small molecule and biologic therapeutics, highlighting previous studies where particle size, shape, and chemistry have been used to enhance systemic particle distribution properties. In addition, we introduce the application of PRINT technology towards respiratory drug delivery, a particular interest due to the pharmaceutical need for increased control over dry powder characteristics to improve drug delivery and therapeutic indices. To this end, we have produced dry powder particles with micro- and nanoscale geometric features and composed of small molecule and protein therapeutics. Aerosols generated from these particles show attractive properties for efficient pulmonary delivery and differential respiratory deposition characteristics based on particle geometry. This work highlights the advantages of adopting proven microfabrication techniques in achieving unprecedented control over particle geometric design for drug delivery.

## 1. Introduction

Particulate drug delivery systems play an important role in the treatment of human disease. Particles such as liposomes, protein nanoparticles, and PLGA microparticles are currently used in marketed drug products using a variety of dosage forms [1, 2]. In particular, particle aerosol inhalation therapy is commonplace for the treatment of respiratory disease. Inhaled therapy using pressurized metered dose inhalers (pMDI), dry powder inhalers (DPI), and nebulizers is an attractive route for treatment of respiratory disease, allowing for local delivery of high concentrations of therapeutics in the lung and avoidance of systemic toxicities associated with oral or injectable therapies [3–6]. Despite the prevalence of aerosol therapy, direct drug delivery to the site of disease remains surprisingly inefficient in part due to the lack of control of particle properties, including particle size, in the drug formulation. Although a wide array of devices are available in the market [7], dose delivery efficiencies for dry powder asthma inhalers range from 3 to 15% for children and 10 to 30% for adults, indicating that less than one third of the contained drug actually reaches the lungs; the most advanced pMDIs deliver only 60% of the inhaled material to central and intermediate bronchial airways [4].

The preparation of respirable particles with reproducible and tunable aerodynamic properties remains a challenge [4, 5]. Conventional fabrication of these pharmaceutical aerosols for DPIs is accomplished by techniques such as micronization (milling) or spray drying [8]. These formulation techniques result in polydisperse aerosol populations, with large particle size distributions and limited control over particle shape. Additional formulation challenges arise with forming dry, nonagglomerating powders comprised of pure active ingredients, especially biologicals like siRNA, proteins, and monoclonal antibodies (mAbs). Indeed, there are currently no marketed dry powder inhaled mAbs or siRNA therapies. The unmet need for improved aerosol drug delivery technologies is large; respiratory diseases including asthma, chronic obstructive pulmonary disease (COPD), cystic fibrosis, and influenza are a significant cause of morbidity and mortality worldwide, with an estimated 10 million lung-disease-related deaths in 2004 globally and with health care costs in the US alone of a projected $173 billion in 2010 [9, 10].

In this work, we demonstrate the use of a top-down, roll-to-roll particle nanomolding technology, (PRINT, Particle Replication in Non-wetting Templates) to fabricate monodisperse, nonspherical particles with unprecedented control over size and shape [11–13] and highlight the benefits that this approach can have for drug delivery and particularly respiratory drug delivery. In addition to new results presented in this paper, we highlight other published studies that demonstrate the breadth and applicability of PRINT drug delivery technology for applications beyond respiratory delivery, including systemic delivery.

In previous efforts, PRINT nanoparticles and microparticles have been used to study the effects of particle size on cellular internalization and particle biodistribution *in vivo*. Gratton et al. studied the effects of particle size and shape on cellular internalization and intracellular trafficking and demonstrated significant dependence on particle size and shape in both the internalization rate and internalization pathways of HeLa cells [14]. Interestingly, the authors demonstrated that rod-like particles show a higher internalization rate than equivalent diameter cylindrical particles. Merkel et al. have examined the role that particle modulus plays in particle circulation *in vivo*, finding that low-modulus hydrogel microparticles have elimination half-lives of greater than 90 hours [15]. Increasing the stiffness of these particles by increasing hydrogel crosslink density can reduce the elimination half-life 30-fold and change the accumulation of these particles from the spleen to the lungs and liver. These two studies highlight the importance that flexible control of particle size, shape, and chemistry affords drug delivery vehicles. Additionally, the PRINT manufacturing process has been demonstrated at scales relevant to support preclinical and clinical studies. Liquidia Technologies has initiated a Phase I clinical study of a PRINT vaccine candidate, demonstrating the production of GMP pharmaceutical materials using this novel nanofabrication process, at a scale relevant to clinical development [16].

The outcome of implementing this particle engineering approach for dry powder fabrication is improved aerosol performance applicable to respiratory drug delivery, demonstrated by incorporation of a variety of pharmaceutically relevant compounds. *In vitro *results demonstrate that PRINT particle aerosols possess high respirable dose, high fine particle fraction, and tunable particle aerodynamic diameter. *In vivo *canine deposition studies demonstrate the ability to influence dry powder delivery as a function of particle geometry. These results suggest that this tunable particle engineering approach is a versatile platform for enabling next-generation respiratory drug delivery. We also highlight some of the utility of PRINT for the production of particles for small molecule, protein, and oligonucleotide drug delivery, which demonstrates that PRINT is a versatile formulation approach and should find applicability in oral, parenteral, and topical dosage forms for multiple disease indications.

## 2. Methods

### 2.1. Fabrication of Particles for Drug Delivery Using PRINT Technology

PRINT is an adaptation of micro- and nanomolding technologies, rooted in the microelectronics industry, that is used to fabricate monodisperse particles of controlled sizes and shapes using roll-to-roll manufacturing processes. It allows for the fabrication of monodisperse particles with precise control over size, shape, composition, and surface functionalization. Unlike many other particle fabrication techniques, the PRINT method is versatile and gentle enough to be compatible with the multitude of next-generation therapeutic and diagnostic agents, including small molecules, protein biologics, siRNA, and bioabsorbable and hydrophilic polymer matrix materials with embedded pharmaceutical cargo.

**Figure 1:**
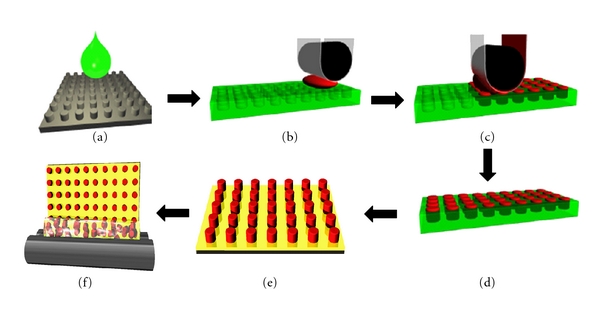
Schematic illustration of the PRINT process. (a) Features on a hard silicon master template are replicated with high fidelity (b) to obtain a soft, polymeric mold with micro- and nanocavities that can then be (c) filled with relevant particle matrix and (d) extracted out of the mold and onto a harvest array for (e) particle collection and purification.

An overview of the PRINT process is outlined in Figure 1. As mentioned previously, the particles produced using the PRINT process are templated using polymeric micromolds. The molds themselves arise from replication of a silicon master template (Figure 1(a)), which is fabricated using advanced lithographic techniques. The replication of the master template results in a precise mold having micro- or nanoscale cavities. Molding of pharmaceutical materials and/or excipients occurs through spontaneous filling of the cavities through capillary forces, with no formation of an interconnecting “flash” layer of material between the cavities (Figures 1(b) and 1(c)). The particles are solidified (Figure 1(d)) and removed from the mold by bringing the mold in contact with an adhesive layer that enables the particles to be easily removed from the mold cavities (Figure 1(e)). At this point free flowing powders or stable dispersions can be obtained by dissolving away the adhesive layer from the particles, with the option to then be further purified, chemically modified, or analyzed (Figure 1(f)). Particles can be used as suspensions or dried using evaporation or lyophilization to produce dry powders.

### 2.2. Fabrication of Particles for Respiratory Drug Delivery

PRINT particles were fabricated and isolated as dry powders as described in previous reports [12, 13, 15, 17, 18]. To highlight the chemical versatility of PRINT particle technology for aerosol delivery of both small molecule and biologic drugs, particles comprised of proteins such as bovine serum albumin (BSA, Sigma-Aldrich) and immunoglobulin G (IgG, Calbiochem), polymers such as poly-lactic-*co*-glycolic acid (PLGA, Mw 30 K, Polysciences), and pharmaceutically relevant compounds such as itraconazole (Spectrum Chemical), zanamivir (Haorui USA), DNase (Worthington Biochemical), and siRNA (Dharmacon) were fabricated. Monodisperse particles from these molds were collected in various aqueous and organic suspensions: for particles consisting of non-water-soluble matrices, such as polymeric and the small molecule itraconazole, distilled water was used to collect the particles from the array; for particles consisting of water-soluble matrices such as zanamivir, DNase, and siRNA, isopropyl alcohol was used to collect the particles from the array. To make porous particles, sacrificial poly(vinylpyrrolidone) porogen are comolded with the drug or drug/excipient blend and selectively removed during the harvesting step. Finally, particles were lyophilized from water or *tert*-butanol in order to obtain dry powder PRINT particles. Itraconazole powder (Spectrum Chemical) was micronized for aerodynamic particle size comparison testing with PRINT particles. Micronization was performed using one pass through the Glen Mills Laboratory Jet Mill.

### 2.3. Chemical and Bioactivity Analyses of Pharmaceutical Compounds in PRINT Particles

PRINT particles composed of small molecules and biologic materials were analyzed to confirm retention of chemical structure and biological activity during the PRINT process. All chromatographic analyses were performed using the Agilent 1100 liquid chromatography system and analyzed in Empower. A gradient reverse-phase high-performance liquid chromatography (RP-HPLC) method for itraconazole analysis was based off of the European Pharmacopoeia (EP) 5.0 method for the compound [19]. Briefly, the chromatographic procedure is a stability-indicating EP method for itraconazole in which the detection has been modified for use with a diode array. This gradient elution method used a Phenomenex Prodigy ODS (3) 100 angstrom, 4.0 × 100 mm, 3 *μ*m analytical column with mobile phase A containing 27.2 g/L tetrabutylammonium hydrogen sulphate in HPLC grade water and mobile phase B containing acetonitrile and used a flow rate of 1.5 mL/min with the following gradient conditions: 0 to 20 min, 20 to 50% mobile phase B; 20 to 25 min, 50% mobile phase B; 25 to 30 min, 20% mobile phase B. Itraconazole was detected with a diode array ultraviolet (UV) measurement at 257 +/− 5 nm with reference background correction at 375 +/− 25 nm and at a retention time of 14.42 minutes.

 A gradient hydrophilic interaction (HILIC)-HPLC method was used for analysis of zanamivir. Briefly, a Waters Atlantic HILIC Silica 5 *μ*m, 4.6 × 100 mm analytical column was used with mobile phase A containing 10 mM ammonium acetate in 1% methanol and 0.05% phosphoric acid in order to maintain a pH of 3 to 4 and mobile phase B containing 0.1% phosphoric acid in acetonitrile. The method used a flow rate of 1.0 mL/min with the following gradient conditions: 0 to 2 min, 80% mobile phase B; 2 to 7 min, 80 to 60% mobile phase B; 7 to 12 min, 60% mobile phase B; 12 to 17 min, 80% mobile phase B. Zanamivir was detected by UV measurement at 230 nm and at a retention time of 5.52 minutes.

A gradient super-anionic-exchange-(SAX-) HPLC method was used for analysis of siRNA. Briefly, a Dionex BioLC DNAPac PA 200 4 × 250 mm analytical column was used with mobile phase A containing 25 mM NaClO_4_ and 10 mM Tris, 20% ethanol and mobile phase B containing 250 mM NaClO_4_ and 10 mM Tris, 20% ethanol, but at a pH of approximately 7.0. The method used a flow rate of 1.0 mL/min with a column temperature of 40 degrees C and the following gradient conditions: 0 to 8 min, 0–100% mobile phase B; 8 to 10 min, 0% mobile phase B. siRNA was detected by UV measurement at 260 nm and had a retention time of 6.37 minutes.

An isocratic size exclusion chromatography (SEC) method was used for analysis of DNase. Briefly, GE Superdex 75 5/150 GL column was used with PBS. The method used a flow rate of 0.3 mL/min, and the protein was detected by UV measurement at 280 nm and at a retention time of 5.14 minutes. In addition to SEC analysis, a DNA-Methyl Green assay was also used to characterize the bioactivity of DNase, as previously performed by others [20]. Briefly, DNA-Methyl Green (Sigma-Aldrich) was solubilized in 0.05 M Tris buffer to a concentration of 0.2 mg/mL. DNase activity, both unprocessed standards (Sigma Aldrich and Worthington) and DNase from PRINT particles, was obtained by adding DNase samples individually to DNA-Methyl Green and measuring the Methyl Green light absorbance at 640 nm at 2 minute intervals. These measurements were used to obtain an initial linear rate of DNA-Methyl Green degradation, which correlates directly to DNase activity.

### 2.4. *In Vitro* Characterization of Particle Size

Aerodynamic particle sizing of all PRINT aerosols was performed using the aerodynamic particle sizer (APS) spectrometer (Model no. 3321, TSI Inc. Shoreview, MN, USA). Dry powder aerosols were dispensed into an aerosol generator using an insufflator device and a volume-calibrated hand pump (Penn Century Inc., PA, USA).

Next-generation impactor (NGI) experiments were used to compare the aerodynamic size distribution of PRINT zanamivir formulations to Relenza. Before testing, NGI stages were coated with silicone oil. To test PRINT formulations, 5 mg of PRINT-zanamivir particles were loaded into a size 3 HPMC capsule, which was loaded into a Monodose device (Plastiape SpA). The loaded Monodose device was attached to an NGI (MSP Model 170) and tested using a 60 L/min flow rate for 4 seconds. Deposited drug was rinsed from the capsule, the device, device adapter, induction port, filter, and each stage of the NGI using 5 to 25 mL HPLC grade water, and the zanamivir content in each rinsate was measured using HPLC and compared to standard curves to determine the absolute weight of zanamivir in the capsule, device, and impactor. Similar methodology was used to measure the aerodynamic particle size distribution of Relenza, with the exception that preseparator stages were used to determine the deposited dose of large (>10 *μ*m) zanamivir/lactose agglomerates.

 Laser diffraction was used to determine the geometric size of micronized itraconazole crystals. Specifically, measurements were performed using a Sympatec HELOS instrument, operated at 5 bar primary pressure and 105 mbar secondary pressure.

### 2.5. Gamma Scintigraphy *In Vivo* Canine Lung Deposition Imaging

Torus aerosols (1.5 *μ*m and 6 *μ*m) for the *in vivo *canine deposition study were fabricated out of a lactose-albumin-leucine blend (64/32/4 mass ratio) and were further labeled with technitium-99 (Tc99m) by isopropyl alcohol coevaporation. Naïve (unlabeled) PRINT particles were mixed with Tc99m in isopropyl alcohol. Ratios of Tc99m : PRINT particle : IPA were held at 50 mCi : 50 mg : 0.75 mL. The mixture was the gently shaken to mix without coating the material on sides of the vials. The mixture was then evaporated under a gentle stream of N_2_. The labeled particles were then immediately loaded into insufflators and used for either validation studies or canine exposures.

In order to confirm the radiolabeling process, the mass median aerodynamic diameter (MMAD) of the materials before and after labeling and the activity median aerodynamic diameter (AMAD) were determined with a next-generation impactor (NGI). The NGI was operated at 30 L/min for all testing. The MMADs of both labeled and naïve aerosols were determined via differential weight analysis of the NGI cups. Following differential weight analysis, the cups were rinsed with 3 mL of water and the water was transferred into a 20 mL scintillation vial. The activity in each cup was quantified with a radio isotope counter. All data were processed to determine the MMAD/AMAD and the geometric standard deviation (GSD) for each aerosol. Based on initial results, it was decided to place a cyclone (URG Corp, model URG-2000-30EC) inline with the aerosol delivery system to remove large agglomerates and achieve an acceptable correlation between the naïve aerosols and Tc99m activity.

 In order to estimate the amount of material dosed using the canine endotracheal exposure system, the delivery system efficiency was first determined for each particle group. This was performed by loading the dry powder reservoir with known amounts of each material (1.5 and/or 6.0 *μ*m torus particles) and collecting aerosolized powder on a filter placed at the exit of the endotracheal tube. The amount of material on the filter and the amount of material delivered from the devices were determined via differential weight analysis. The delivery efficiency was calculated as the percentage of material delivered from the dry powder reservoir device that exits the endotracheal tube and is ultimately available to the lower respiratory tract.

At the time of exposure, multiple dry powder reservoirs were loaded to target an aerosol delivery of 10 mCi and ensure sufficient Tc99m deposition in the canine lungs for image analysis. Prior to being exposed, animals were placed on isofluorane anesthesia and apnea was induced by hyperventilation. Immediately following the aerosol exposures, the endotracheal tube was removed and the dogs were transferred to the Siemens E.Cam clinical SPECT gamma camera and a 10 minute planar gamma image was collected. The time lapsed from the start of aerosol exposures until the start of imaging was ~1.5 to 2 minutes, and the time from the start of aerosol exposures until the completion of the imaging was typically ~12 minutes. During image acquisition, the dry powder reservoirs were quantified for radioactivity to determine the amount of activity aerosolized. This value was then multiplied by the predetermined delivery efficiency in order to estimate the lower respiratory tract dose, or dose presented at the exit of the endotracheal tube, for each experiment.

### 2.6. Canine Lung Deposition Image Analysis

Image analysis was performed with the Siemens ICON software to determine the activity in two canine regions of interest (ROI) for each animal: the lungs and the trachea. In order to correlate the counts in each ROI to activity, a standard curve was prepared for the gamma camera to define the relationship between activity (measured with a radioisotope counter) and counts (from the image analysis). After converting measured counts to radioactivity, the quantified amount of activity in the lung ROI was then divided by the quantified amount of activity in the lung ROI in order to determine whole lung deposition counts normalized to trachea counts for each animal. Statistical differences in this measurement were evaluated by paired, two-tailed *t*-test across the four animals used for lung deposition imaging.

## 3. Results

### 3.1. Precisely Engineered Particles Containing Pharmaceutically Relevant Components

**Figure 2:**
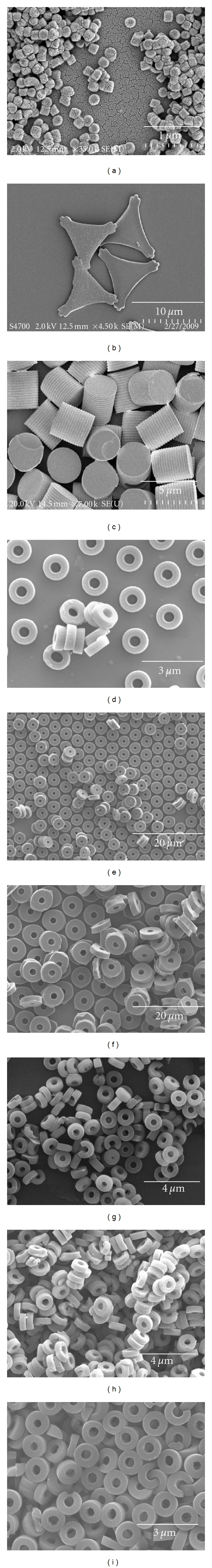
SEM micrographs of diverse PRINT aerosols. (a) BSA/Lactose 200 × 200 nm cylinders; (b) IgG/Lactose10 *μ*m pollen; (c) 30 K PLGA 3 *μ*m cylinders; (d) itraconazole 1.5 *μ*m torus; (e) itraconazole 3 *μ*m torus; (f) itraconazole 6 *μ*m torus; (g) zanamivir 1.5 *μ*m torus; (h) DNAse 1.5 *μ*m torus; (i) siRNA 1.5 *μ*m torus.

To illustrate the delivery of relevant therapeutic compounds to the respiratory tract, we fabricated particles with independent control of particle size, shape, and composition. An array of SEM micrographs is shown in Figure 2 highlighting PRINT's versatility: BSA/lactose blend 200 × 200 nm cylinders (Figure 2(a)); IgG/lactose blend 10 *μ*m “pollen” (Figure 2(b)); poly-lactic-*co*-glycolic acid (PLGA, Mw 30 K) 3 *μ*m cylinders (Figure 2(c)); itraconazole (marketed as Sporanox for treatment of fungal infection) molded into 1.5 *μ*m, 3 *μ*m, and 6 *μ*m torus particles (Figures 2(d)–2(f)); 1.5 *μ*m torus particles comprised of pharmaceutically relevant compounds including zanamivir (marketed as Relenza for treatment of influenza) (Figure 2(g)); bovine DNase (recombinant human DNase is marketed as Pulmozyme for treatment of cystic fibrosis) (Figure 2(h)); siRNA (Dharmacon) (Figure 2(i)). The “pollen” shape in Figure 2(b) is a biomimetic design, based on the shape of the pollen *Eperua schomburgkiana*.

In order to confirm that the PRINT particle fabrication process used to generate engineered aerosols did not alter the chemical structure of pharmaceutical compounds, analytical tests were performed to determine the compound integrity following fabrication as compared to the unprocessed or reference compound. Purity of compounds in PRINT particles relative to unprocessed or reference compound was measured to be 99.6% for itraconazole (RP-HPLC), 100% for zanamivir (HILIC-HPLC), 99.2% for siRNA (SAX-HPLC), and 99.0% for DNase (SEC). Additionally, IC_50_ in DNA-Methyl Green assay yielded DNase IC_50_ values for reference DNase (Worthington) and PRINT-DNase of 26.5 and 18.8 Kunitz units/mL, respectively, indicating that PRINT particle fabrication does not alter DNase bioactivity.

### 3.2. Aerodynamic Characteristics of PRINT Aerosols

**Figure 3:**
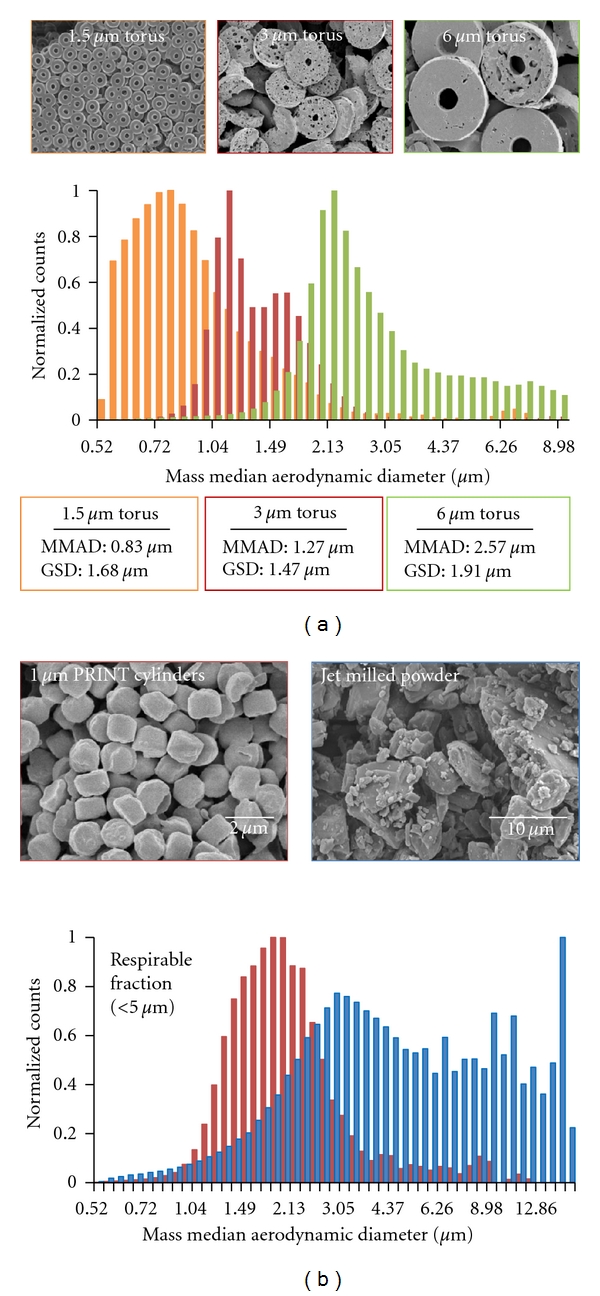
Aerodynamic characterization of PRINT aerosols. (a) SEM micrographs and aerodynamic performance of 1.5 *μ*m, 3 *μ*m, and 6 *μ*m particles by APS. PRINT affords precise control over particle geometric size and aerodynamic size. (b) SEMs and aerodynamic distributions of jet-milled itraconazole aerosols compared to 1 *μ*m PRINT cylinder particles made out of itraconazole. PRINT-itraconazole particles result in a narrower size distribution and higher available respirable fraction.

Physical characterization of PRINT aerosols confirmed the ability to produce highly dispersible aerosols with controllable and narrow aerodynamic size distributions.  Figure 3(a) demonstrates the capability to tune particle aerodynamic size on the basis of particle design. We fabricated torus particles with geometric sizes 1.5 *μ*m, 3 *μ*m, and 6 *μ*m torus and measured their aerodynamic characteristics using a time-of-flight aerodynamic particle sizer (APS). For these particles, porogen was added to the formulation, then subsequently removed to produce porous particles. The mass median average aerodynamic diameters (MMAD) of these particles were measured to be of 0.83 *μ*m, 1.27 *μ*m, and 2.57 *μ*m with geometric standard deviations (GSD) of 1.68 *μ*m, 1.47 *μ*m, and 1.91, respectively.

To compare the size distributions of PRINT aerosols to conventional fabrication techniques (Figure 3(b)), we compared the mass-weighted aerodynamic particle size distribution (mass median aerodynamic diameter, MMAD) of 1.5 *μ*m PRINT cylinders composed of itraconazole to the particle size distribution of jet-milled itraconazole (geometric size **×**10 = 0.77 *μ*m; **×**50 = 2.79 *μ*m; **×**90 = 7.42 *μ*m). Jet milling is the most commonly utilized technique for preparation of respirable aerosol particles. The PRINT aerosol had a narrower distribution and a higher fraction of drug in the respirable range (less than 5 *μ*m), indicating that the aerodynamic properties of these particles are better suited for inhalation therapies. Moreover, according to well-accepted correlations of aerodynamic particle size and lung deposition, it can be expected that the 1 *μ*m cylinder particles will have enhanced deposition in peripheral airways (alveoli and respiratory bronchioles) compared to the larger particles. The precise control over aerodynamic size of PRINT aerosols may be clinically useful for local drug delivery to the lungs by enhancing deposition efficiency at the site of disease and limiting unintended off-target effects [21].

### 3.3. Engineered PRINT Aerosols Exhibit Increased Aerosol Delivery *In Vitro*

**Figure 4:**
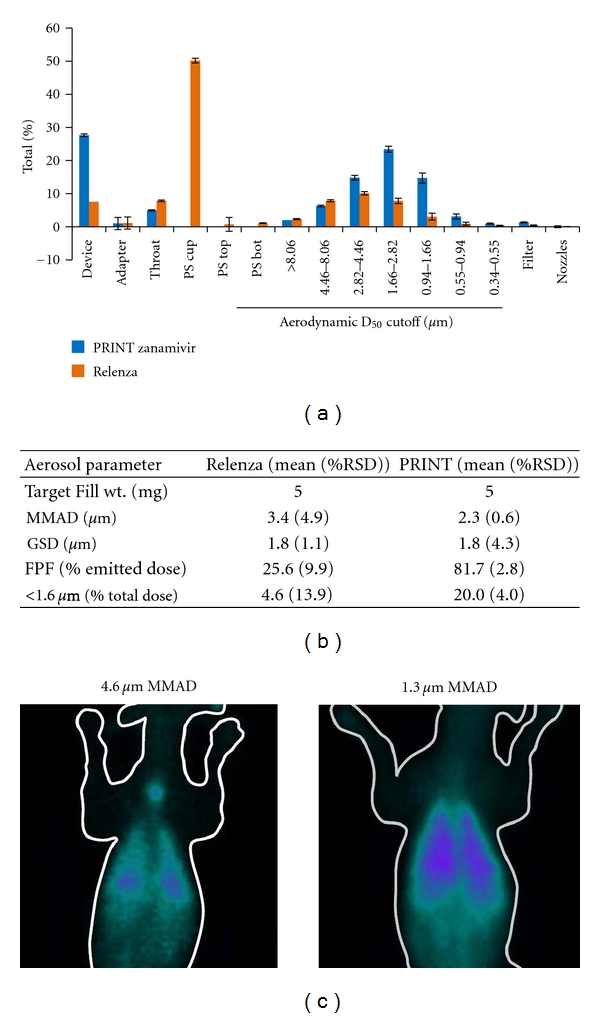
Favorable properties of PRINT aerosols for dry powder pharmaceutical use. (a, b) Comparison of 1.5 *μ*m torus PRINT-zanamivir particles against the marketed product Relenza (active pharmaceutical ingredient zanamivir) using an NGI. (b) PS: preseparator; RSD: relative standard deviation. (c) Whole lung deposition by gamma scintigraphy in canine shows increased whole-lung deposition of 1.5 *μ*m (right, 1.3 *μ*m MMAD) torus aerosols versus 6.0 *μ*m (left, 4.6 *μ*m MMAD) torus aerosols.

We compared the *in vitro* performance of pharmaceutically relevant PRINT particle aerosols to a dry powder marketed product. This was carried out using Relenza (GlaxoSmithKline), a small molecule DPI indicated for treatment of influenza, which contains the active pharmaceutical ingredient, zanamivir (5 mg), blended with micronized lactose (20 mg). 1.5 *μ*m torus PRINT-zanamivir formulations were prepared, directly packaged into capsules, and aerosolized from a low-resistance DPI device (Monodose, Plastiape SpA). Both PRINT-zanamivir and Relenza formulations were characterized with a next-generation impactor (NGI). As shown in Figures 4(a) and 4(b), the PRINT-zanamivir formulation resulted in significantly improved delivery compared to Relenza. For the same fill weight (5 mg), the PRINT zanamivir dosage form showed a smaller MMAD, a similar GSD, 3 to 4 times higher fine particle fraction (FPF) and respirable dose, and 4 to 5 times more deposition of material in the size range of less than 1.6 *μ*m. It is expected that the device retention of the PRINT-zanamivir formulation could be significantly decreased with tuning of the fill weight or device characteristics, which is beyond the scope of the work presented here. These results indicate that finer engineered PRINT particles should correlate to superior drug delivery to the lower respiratory tract (Figure 4(b)). Based on literature studies of the deposition patterns of Relenza in healthy human volunteers, it is known that 77% of the emitted drug from the commercial product is deposited in the oropharynx rather than the lung [22]. Thus, the *in vitro* results presented here suggest that the PRINT-zanamivir aerosol would translate to significantly more efficient lung delivery compared to Relenza.

### 3.4. PRINT Aerosols with Narrow Size Distributions Exhibit Distinct *In Vivo* Lung Deposition Patterns

Finally, we demonstrated the ability of PRINT particle aerosols to control *in vivo* pulmonary delivery using a canine deposition model. PRINT aerosols composed of lactose, albumin, and leucine (64/32/4 mass ratio) were prepared, radiolabeled with technetium-99, and aerosolized into the respiratory tract of beagle dogs using an endotracheal dosing apparatus. As shown in the gamma scintigraphic images (Figure 4(c)), significantly more whole-lung deposition was achieved with 1.5 *μ*m versus 6 *μ*m torus particles (1.3 *μ*m and 4.6 *μ*m MMAD, resp.), as would be expected from the relative aerodynamic sizes of these particles. Image analysis and quantification of the radioactivity counts confirmed this observation. In addition, the torus 1.5 *μ*m particles showed a greater than twofold enhancement of whole-lung deposition counts normalized to trachea deposition. This ability to tailor particle lung deposition could have broad applicability for respiratory drug delivery, particularly in scenarios where peripheral lung deposition should be enhanced or avoided depending on clinical application.

## 4. Discussion

The PRINT fabrication approach predictably controls particle geometric and aerodynamic features, a differentiating attribute as compared to traditional particle generation approaches. In particular, micromolding strategies such as PRINT represent one of the only methods to precisely control particle shape and size. For PRINT, the particle geometry is directly derived from the semiconductor wafer, bringing inherent nanoscale precision to the particle geometry and offering the capability to generate unique, nonspherical shapes. It is possible to control geometric features such as length, aspect ratio, and edge curvature, as well as adding unique features such as fenestrations and biomimetic designs, as shown in Figure 2. The capability of PRINT to prepare micro- and nanoparticles of a diverse set of materials is due to the ability to mold materials in a variety of physical forms. In addition to the detailed studies presented here, particles have been prepared by polymerization [11] or solvent evaporation [23]. This flexibility lends itself to the preparation of pharmaceutically relevant particles such as hydrogels [15], PLGA controlled-release systems [13], stimuli-responsive particles [17], suspension formulations [14], or dry powder aerosols as presented here (Figures 2 and 3). This ability to control particle size, shape, and uniformity should also find advantageous use in many dosage forms, including oral, topical, and parenteral products. 

Microfabrication techniques such as PRINT offer the advantage of deterministic control of particle geometry that is inherent from the use of semiconductor manufacturing techniques. In the case of PRINT technology, the same master template can be used to create each batch of micromolds and particles for a particular size and shape. Thus, each batch of particles possesses high uniformity and batch-to-batch consistency, regardless of the batch size. In addition, the uniform particle populations that are produced lend themselves to straightforward in-process characterization using a number of standard particle sizing methods, such as microscopy and light scattering. These features make the PRINT technology attractive from the perspective of compliance with Quality-by-Design directives from the FDA.

From a formulation perspective, PRINT technology has been shown to be a versatile approach to deliver many classes of therapeutic compounds and excipients. Particle size can be controlled over several orders of magnitude, from the sub-100 nm scale to hundreds of microns. In traditional fabrication methods, particle chemical composition and physical characteristics such as geometric or aerodynamic size are inherently coupled, for example, the molecular properties of a small molecule pharmaceutical ingredient are known to impact the particle size distribution of micronized particles, whereas the solubility and drying kinetics of precursor solutions can impact the particle size distribution of spray-dried particles [8]. In contrast, micromolded particle engineering has the ability to define the particle size and shape independent of the input material properties, which was demonstrated by fabricating particles of identical geometry yet comprising hydrophilic and hydrophobic small molecules, proteins, or nucleic acids (Figures 2(d)–2(i)). While particularly relevant for aerosol lung delivery, this ability to independently control particle composition and physical size should find utility in multiple dosage forms and routes of administration.

Small molecule drug compounds can be formulated as drug alone or drug/excipient mixtures with tunable loading. Enlow et al. demonstrated the production of PLGA/docetaxel PRINT nanoparticles with up to 40% chemotherapeutic loading [13]. This finding is in contrast to typical polymer nanoparticle drug delivery systems produced by emulsion [24], nanoprecipitation [25], and ultrasonication [26] that have theoretical drug loading of less than 15% and variable encapsulation efficiency. Furthermore, the authors demonstrated the ability to independently tune particle size, shape, and drug loading. *In vitro* results indicated that potency of these PLGA-docetaxel nanoparticles was up to 10x greater than Taxotere, a commercially marketed micellar formulation of docetaxel. In this work, we highlight the ability of PRINT to fabricate particles of neat small molecule drugs. Figures 2(d)–2(f) show particles composed of 100% itraconazole, prepared by molding an amorphous itraconazole glass. Particles composed of zanamivir were also fabricated (Figure 2(g)), and both itraconazole and zanamivir particles showed good aerosol delivery performance *in vitro* (Figures 3 and 4).

PRINT particles can be prepared from protein and oligonucleotide therapeutic agents as well. Kelly and DeSimone demonstrated the capability to use PRINT technology to fabricate monodisperse particles of albumin and insulin without causing agglomeration of the protein [12]. In this work, we demonstrate molding of DNase, a therapeutic protein for cystic fibrosis (marketed as Pulmozyme). Figure 2(h) shows 1.5 *μ*m torus particles composed of DNase. Size exclusion chromatography of PRINT-DNase microparticles shows minimal agglomeration of the protein, and *in vitro* bioassay measurements demonstrate equivalent enzyme activity to naïve DNase. Oligonucleotide molecules such as siRNA therapeutics were also successfully molded as particles (Figure 2(i)) with retention of chemical structure. Taken together, these data demonstrate that PRINT particles can be formed of biological materials without aggregating/denaturing the molecule or changing its functionality.

Micromolded particles produce high-performance aerosols that possess tunable aerodynamic diameters and narrow aerodynamic size distributions. This control over aerosol characteristics was demonstrated across a wide range of aerodynamic diameters within the respirable range (Figure 3(a)) and through differential *in vivo* lung deposition based on particle size (Figure 4(c)). In addition, PRINT aerosols achieve an increased respirable dose and decreased MMAD, including the dose fraction below 1.6 *μ*m, compared to aerosols generated by traditional micronization processes (Figures 3(b) and 4(a)). These attributes are expected to translate into more efficient respiratory drug delivery for a wide range of therapeutics that are intended to deposit in the lung periphery. Importantly, the aerosolization of PRINT particle dry powders does not require the use of bulking excipients, such as lactose, for particle dispersion, as is often the case for dry powder products. Elimination of bulking agents potentially simplifies the chemistry, manufacturing, and control processes required to develop dry powder products, as well as mitigating the potential for excipient-induced user side effects.

The micromolding particle fabrication approach presented here also holds the potential to engineer dry powder aerosols optimized for specific disease targets. There are a number of instances where more precise respiratory drug delivery could be useful, as has been demonstrated by others. Particle aerodynamic size and regional drug deposition has been shown to influence pharmacodynamic responses in diseases such as asthma and cystic fibrosis. Usmani et al. demonstrated that 6.0 *μ*m MMAD albuterol aerosols improve forced expiratory volume (FEV1) in asthmatic subjects to a greater degree than 3 *μ*m or 1.5 *μ*m aerosols. The authors correlated the enhancements FEV1 to higher central lung deposition (confirmed by scintigraphy) and postulated that the pharmacodynamic advantage of these 6.0 *μ*m aerosols was related to greater deposition in proximity to conducting airway smooth muscle tissue [27]. In another study in cystic fibrosis patients, improved forced expiratory fraction (FEF_75_) was observed for DNase aerosols delivered preferentially to the small airways compared to the large airways. This data suggests that enhanced deposition of DNase at the site(s) of disease pathology could benefit patient lung function [28]. In addition, it is reasonable to expect that enhanced deposition in the alveolar region may be favorable for applications such as systemic delivery of therapeutics via the lung [21]. These studies suggest that technologies such as PRINT, which possess the ability to engineer particles with desirable aerosol and deposition characteristics, could ultimately result in inhaled products with enhanced efficacy when applied to the appropriate disease and therapeutic compound. In particular, the benefits of differential lung deposition and efficient lung delivery will be particularly useful for expensive therapeutic agents such as biologics or highly potent, narrow therapeutic index compounds.

Lastly, particle shape is known to influence all stages of pulmonary drug delivery: from entrainment and deagglomeration into a disperse aerosol [21, 29, 30], to aerodynamic characteristics and deposition [8, 30–34], to mucociliary clearance and macrophage uptake [14, 35, 36]. Others have demonstrated that shape has an impact on particle aerodynamic characteristics through studies on simple shapes, such as rods, plates, fibers, and spheres [30, 31]. Though particle shape is known to be a critical factor of aerosol properties, thorough exploration of its effect has been limited by current fabrication methods of aerosol particles [31]. Controlling particle shape thus provides an opportunity to systematically optimize the effect of shape on these stages of drug delivery. Microfabrication techniques such as PRINT offer a promising strategy to control particle shape, and more thorough investigations on the impact of particle shape on lung deposition, clearance, and cellular internalization are currently underway in order to better characterize the specific benefits particle shape may hold for respiratory drug delivery.

## 5. Conclusion

In summary, coopting the top-down manufacturing capabilities of the microelectronics industry enables the generation of high-precision particle-based drug delivery systems that are compatible with novel and existing formulation strategies and dosage forms. In particular, the PRINT process is well suited for the production of high-performance aerosol particles for respiratory drug delivery. Precise control over size and shape allows for defined aerodynamic properties, which, in turn, leads to enhanced aerosol performance and differential lung deposition *in vivo*. In addition to the benefits imparted by control over particle size and shape, micromolding is presented as a versatile strategy for formulating particle systems of small molecules, biologics, oligonucleotides, and drug/excipient mixtures. Overall, micro-molding is a viable particle design strategy that may address challenges existing for respiratory drug delivery and other dosage forms, thereby constituting a promising opportunity for the development of next-generation therapeutics.
